# Factors related to cardiac rupture after acute myocardial infarction

**DOI:** 10.3389/fcvm.2024.1401609

**Published:** 2024-10-02

**Authors:** Xue Gao, Ying Guo, Xiaoting Zhu, Chunlei Du, Beibei Ma, Yinghua Cui, Shuai Wang

**Affiliations:** Department of Cardiology, Affiliated Hospital of Jining Medical University, Jining Medical University, Jining, Shandong, China

**Keywords:** acute myocardial infarction, risk factors, cardiac rupture, in-hospital mortality, prognosis

## Abstract

**Background:**

Cardiac rupture (CR) after acute myocardial infarction (AMI) is a fatal mechanical complication. The early identification of factors related to CR in high-risk cases may reduce mortality. The purpose of our study was to discover relevant risk factors for CR after AMI and in-hospital mortality from CR.

**Methods:**

In this study, we enrolled 1,699 AMI cases from October 2013 to May 2020. A total of 51 cases were diagnosed with CR. Clinical diagnostic information was recorded and analyzed retrospectively. We randomly matched these cases with AMI patients without CR in a 1:4 ratio. Univariate and multivariate logistic regression and stratifying analysis were used to identify risk factors for CR. Univariate and multivariate Cox regression hazard analysis and stratifying analysis were used to assess predictors of in-hospital mortality from CR.

**Results:**

The incidence of CR after AMI was 3.0% and in-hospital mortality was approximately 57%. Multivariate logistic regression analysis identified that white blood cell count, neutrophil percentage, anterior myocardial infarction, a Killip class of >II, and albumin level were independently associated with CR (*p* < 0.05). Stratifying analysis showed that age, systolic blood pressure, and bicarbonate were independent risk factors for female CR (*p* < 0.05) but not male CR. Triglyceride and cardiac troponin I were independent risk factors for male CR (*p* < 0.05) but not female CR. Anterior myocardial infarction, a Killip class of >II, and neutrophil percentage were independent risk factors for male and female CR (*p* < 0.05). Multivariate Cox regression analysis showed that the time from symptom to CR and the site of CR were independent predictors for in-hospital mortality from CR (*p* < 0.05). Stratification analysis indicated that risk factors did not differ based on gender, but platelet counts were predictors for in-hospital mortality in female and male CR.

**Conclusion:**

Low albumin, a high white blood cell count, neutrophil percentage, anterior myocardial infarction, and a Killip class of >II were independent and significant predictors for CR. However, risk factors are different in male and female CR. The time from symptom to CR, the site of CR, and platelet counts were independent predictors for in-hospital mortality from CR. These may be helpful in the early and accurate identification of high-risk patients with CR and the assessment of prognosis. In addition, gender differences should be considered.

## 1 Introduction

Acute myocardial infarction (AMI) is a major cause of global mortality. Cardiac rupture (CR) is a dramatic and potentially lethal mechanical complication (MC) after AMI (1, 2). According to the onset time, there are three types of CR: acute, subacute, and chronic (3). CR is further classified by location: ventricular septal rupture (VSR), papillary muscle rupture (PMR) and dysfunction, free wall rupture (FWR), and left ventricular (LV) pseudoaneurysm formation (4). Electrolysis and hemodynamic instability are closely related to wall rupture (5). Sudden cardiac death and cardiogenic shock are the main clinical features of ruptured infarcted myocardial tissue (6). Studying and treating CR are made difficult by its sudden onset. Some predisposing factors such as being female, being of an advanced age, and having no previous history of MI are highly correlated with CR (7). However, the relationship between hypertension and CR is controversial (8). Over the past 30 years, the incidence and mortality of cardiac rupture appeared to have declined with reperfusion strategies and adjuvant drug therapy (7). Nevertheless, it remains difficult to predict the occurrence of CR. The purpose of our study was to discover relevant risk factors for predicting CR after AMI and in-hospital mortality from CR.

## 2 Methods

### 2.1 Study design

**Figure 1:**
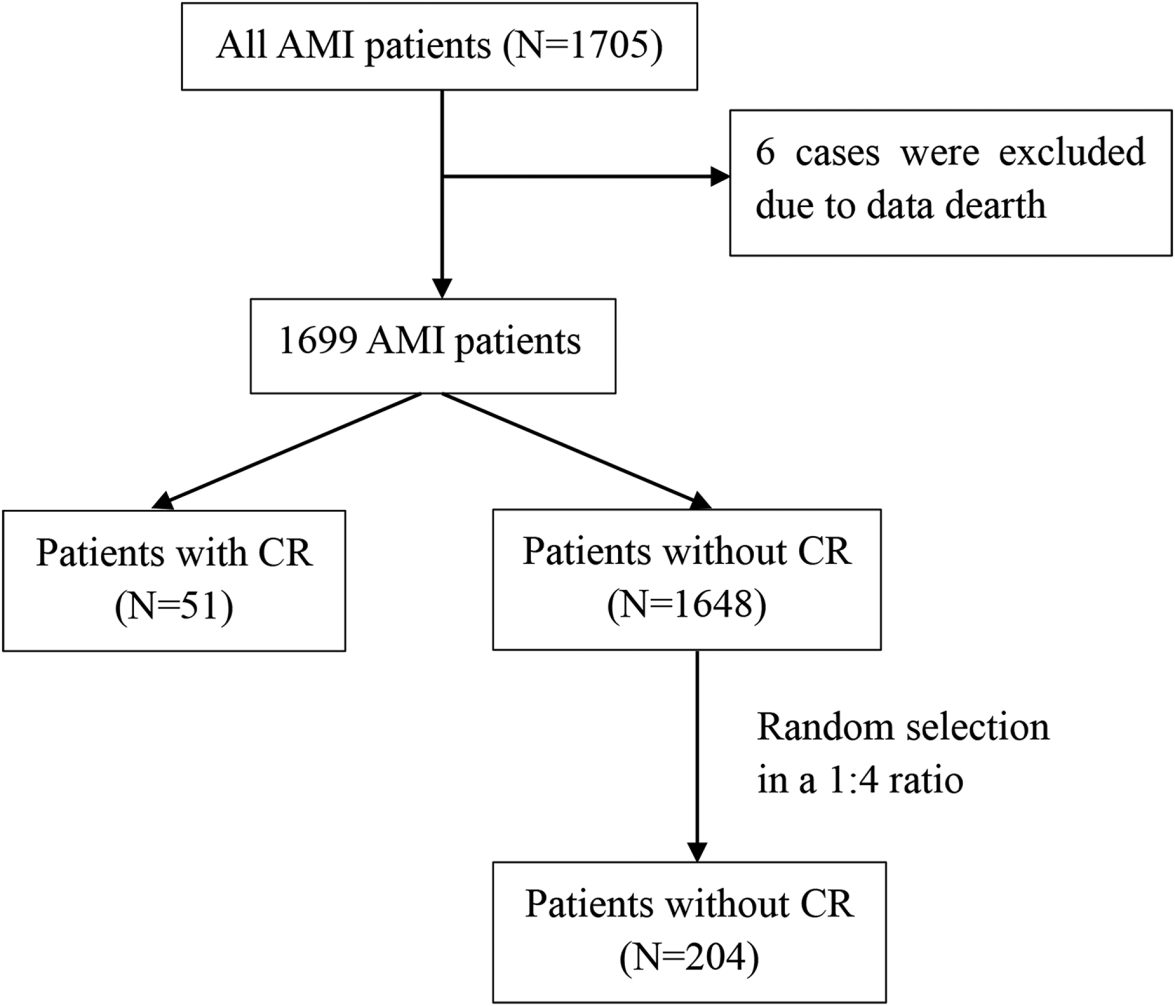
Flow diagram of the case selection. AMI, acute myocardial infarction; CR, cardiac rupture.

We performed a single-center retrospective study. The study screened 1,705 patients with a diagnosis of AMI admitted to the cardiology department of the Affiliated Hospital of Jining Medical University between October 2013 and May 2020. A total of 1,699 patients were included in this analysis, after excluding 6 patients with missing data. The flow diagram of the case selection is shown in Figure 1. In the present study, data were compiled from the hospital's electronic medical record system. The study was approved by the Human Ethics Committee of the Affiliated Hospital of Jining Medical University, with a waiver for informed consent.

### 2.2 Study population

A total of 1,699 patients hospitalized with AMI were recruited. Fifty-one patients (3.0%) developed CR, including 19 with FWR, 25 with VSR, and 7 with PMR.

The diagnostic criteria of AMI, including ST-segment elevation myocardial infarction (STEMI) and non-STEMI, were as follows: (1) elevation of cardiac biomarkers [creatine kinase-MB (CK-MB) or troponin I or T], (2) typical chest pain with a sudden onset and long persistent period, (3) a new regional wall motion abnormality with imaging evidence, and (4) ST-segment elevation of ≥0.2 mV or depression, prominent T-wave inversion, or pathological Q waves with an ECG.

The diagnostic criteria of CR were as follows: (1) FWR was proven by echocardiography or a large pericardial effusion with pericardiocentesis once AMI patients experienced sudden cardiogenic shock or electrical mechanical dissociation, (2) PMR was confirmed by new mitral regurgitation with echocardiography, and (3) VSR was identified by new significant left-to-right shunting on echocardiography and a cardiac systolic murmur. We collected the clinical data of patients from the hospital's electronic medical record system, including characteristics, medical history, and laboratory parameters, etc.

### 2.3 Statistical analysis

The Kolmogorov–Smirnov goodness-of-fit test was performed to test the normality of continuous data. Normally distributed continuous variables are represented by mean ± SD and were obtained using an independent sample *t*-test. Continuous variables with skewed distributions are expressed by medians (with interquartile ranges) and were obtained using rank-sum testing. Categorical variables are expressed as percentages, and comparisons were performed using Pearson’s chi-squared test or Fisher's exact test. We randomly selected 204 AMI patients without CR in a 1:4 ratio as the control group. Factors associated with CR were assessed using binary logistic regression, and the univariate model was further adjusted for the variables age and sex. Subsequently, multivariate analysis was carried out to analyze risk factors with *p* < 0.1 in the univariate analysis results or clinically significant factors to assess independent predictors of CR. Univariate and multivariable Cox regression hazard analysis was used to estimate the factors associated with in-hospital mortality from CR. Stratifying analysis based on sex was further used to eliminate the interference of confounding factors. All *p*-values were two-tailed; *p* < 0.05 was considered statistically significant. SPSS v25.0 statistical software was used.

## 3 Results

### 3.1 Clinical characteristics of AMI patients with CR

**Figure 2:**
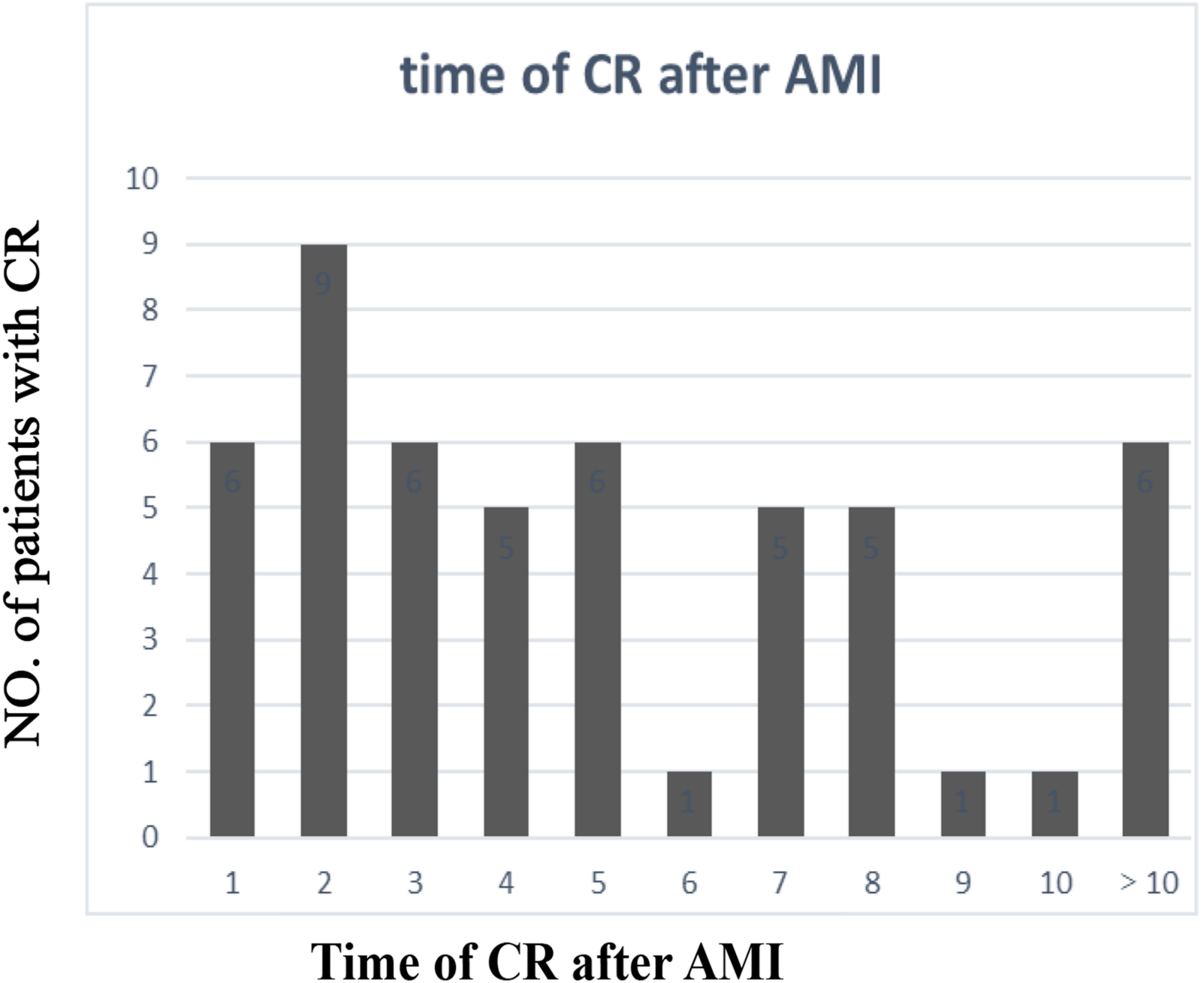
Time of CR after AMI. CR, cardiac rupture; AMI, acute myocardial infarction.

The clinical characteristics of the study population are presented in Supplementary Table 1. Significant differences in age, gender, the body mass index (BMI), systolic blood pressure (SBP), the left ventricular ejection fraction (LVEF), the cardiac troponin I (cTnI) level, the myoglobin (MYO) level, the glucose level, white blood cell (WBC) counts, red blood cell (RBC) counts, the hemoglobin (Hb) level, the neutrophil percentage (neut%), the albumin level, the creatinine (Cr) level, the lactate dehydrogenase (LDH) level, the triglyceride (TG) level, the site of AMI, and the Killip class were detected in the CR and no-CR groups (*p* < 0.05). We further separated by gender and examined the difference in clinical characteristics between the CR and no-CR groups. The significant results are detailed in Table 1 (see Supplementary Table 2 for full results). Among the female patients, compared with the no-CR group, CR patients had lower diastolic blood pressure (DBP), lower SBP, and lower total protein (TP) levels (*p* < 0.05) but higher blood glucose levels (*p* < 0.05). Among the male patients, CR patients were older and had a lower BMI, lower RBC counts, a lower Hb level, and a lower TG level than no-CR (*p* < 0.05) but a higher Cr level (*p* < 0.05). We established that male CR patients had higher WBC counts, neut%, cTnI, and MYO levels but lower albumin levels (*p* < 0.05) than male no-CR patients, with similar results established in females. Combined with Table 2, we found that primary AMI patients were more common in the CR patient group, accounting for 95.5% of female patients and 93.1% of male patients, but this difference was not significant. The majority of infarction sites were anterior in the CR group, making up 63.6% of female patients and 62.1% of male patients (*p* < 0.05). Echocardiography showed a lower LVEF in the CR group with the worst Killip class on arrival. The difference in the LVEF between CR and no-CR was statistically significant in males (*p* < 0.05) but not in females. The Killip class between CR and no-CR had significant differences in both males and females (*p* < 0.05). The time delay from AMI symptom onset to hospital admission was more common; therefore, only 13 (25%) CR patients received percutaneous coronary intervention (PCI) treatment. The mean days from AMI symptom onset to CR was 5.07 ± 7.18 days in males and 2.39 ± 2.56 days in females. CR was most common in the first 3 days after AMI symptom onset (Figure 2). Only four (7.8%) patients underwent surgical repair. Of the 51 patients with CR, 29 (57%) died in the hospital.

**Table 1:** Characteristics of CR and no-CR patients in females and males.

Variables	Female (*n* = 507)		*p*-value
	No-CR (*n* = 485)	CR (*n* = 22)	
SBP (mmHg)	127.13 ± 25.00	106.73 ± 23.40	<0.001
DBP (mmHg)	75.00 (67–86)	64.50 (55.75–79.00)	0.010
cTnI (ng/ml)	2.06 (0.39–10.03)	14.45 (6.11–48.43)	<0.001
MYO (ng/ml)	113.10 (38.95–321.55)	277.81 (78.47–550.29)	0.026
Glucose (mmol/L)	6.50 (5.20–9.60)	7.85 (6.58–10.93)	0.029
WBC (×10^9^/L)	9.60 (7.21–11.90)	12.42 (10.94–16.83)	<0.001
Neut% (%)	77.40 (69.50–83.60)	82.43 (73.97–87.70)	0.050
TP (g/L)	63.45 ± 6.17	60.73 ± 6.51	0.044
Albumin (g/L)	37.68 ± 4.03	35.72 ± 5.16	0.028
Site of AMI, *n* (%)			0.001
Anterior	164 (66.2%)	15 (68.2%)	
No-anterior	321 (33.8%)	7 (31.8%)	
Killip class, *n* (%)			<0.001
≤II	439 (90.5%)	14 (63.6%)	
>II	46 (9.5%)	8 (36.4%)	
Variables	Male (*n* = 1,192)		*p*-value
	No-CR (*n* = 1,163)	CR (*n* = 29)	
Age (years)	62 (53–69)	70 (64–76)	<0.001
BMI (kg/m^2^)	24.80 (22.50–27.04)	23.37 (20.95–25.11)	0.023
LVEF (%)	50.00 (44–54)	46.00 (38.50–50.00)	0.002
cTnI (ng/ml)	2.31 (0.17–12.37)	17.68 (3.28–49.72)	<0.001
MYO (ng/ml)	105.08 (41.90–330.06)	225.66 (79.19–499.01)	0.024
WBC (×10^9^/L)	9.80 (7.72–12.03)	13.38 (10.90–15.76)	<0.001
RBC (×10^12^/L)	4.52 (4.18–4.84)	4.34 (3.60–4.65)	0.024
Hb (g/L)	140.00 (129.00–150.00)	129.00 (113.00–147.00)	0.008
Neut% (%)	76.20 (69.60–82.34)	85.74 (77.00–88.41)	<0.001
Albumin (g/L)	38.64 ± 3.85	35.89 ± 4.60	<0.001
Cr (µmol/L)	68.20 (60.30–79.00)	83.80 (69.60–118.40)	<0.001
TG (mmol/L)	1.20 (0.85–1.69)	0.81 (0.62–1.07)	<0.001
Site of AMI, *n* (%)			0.001
Anterior	446 (38.3%)	20 (69.0%)	
No-anterior	717 (61.7%)	9 (31.0%)	
Killip class, *n* (%)			<0.001
≤II	1,079 (93.8%)	18 (62.1%)	
>II	84 (7.2)	11 (37.9%)	

CR, cardiac rupture; DBP, diastolic blood pressure; SBP, systolic blood pressure; cTnI, cardiac troponin I; MYO, myoglobin; WBC, white blood cell; Neut%, neutrophil percentage; TP, total protein; AMI, acute myocardial infarction; BMI, body mass index; LVEF, left ventricular ejection fraction; RBC, red blood cell; Hb, hemoglobin; Cr, creatinine; TG, triglyceride.

**Table 2:** The clinical characteristics of CR patients, stratified by gender.

Variables	Female (*n* = 22)	Male (*n* = 29)	*p*-value
Primary AMI, *n* (%)	21 (95.5%)	27 (93.1%)	1.000
Site of AMI, *n* (%)			—
One wall	16 (72.7%)	24 (82.8%)	
Anterior	14 (63.6%)	18 (62.1%)	
Inferior	2 (9.1%)	5 (17.2%)	
Posterior	0	1 (3.5%)	
Two walls or more	6 (27.3%)	3 (10.3%)	
NST	0	2 (6.9%)	
Killip class, *n* (%)			0.909
≤II	14 (63.6%)	18 (62.1%)	
>II	8 (36.4%)	11 (37.9%)	
Site of CR			0.491
VSR	10 (45.5%)	15 (51.7%)	
PMR	2 (9.1%)	5 (17.2%)	
FWR	10 (45.5%)	9 (31.0%)	
White blood cell count (×10^9^/L)	13.27 ± 4.09	16.14 ± 13.31	0.530
Time from AMI to admission (days)	2.39 ± 2.56	5.07 ± 7.18	0.321
Patients, <12 h, *n* (%)	7 (31.8%)	7 (24.1%)	0.543
Patients, ≥12 h, *n* (%)	15 (68.2%)	22 (75.9%)	
PCI, *n* (%)			0.297
No	18 (81.8%)	20 (69.0%)	
Yes	4 (18.2%)	9 (31.0%)	
CABG, *n* (%)			—
No	0	26 (89.7%)	—
Yes	0	3 (10.3%)	—
Time from AMI to CR (days)	3.85 ± 2.71	7.10 ± 7.14	0.116
Surgical operation, *n* (%)	0	4 (13.8%)	—
In-hospital outcome, *n* (%)			0.395
Death	14 (63.6%)	15 (51.7%)	
Survive	8 (36.4%)	14 (48.3%)	

AMI, acute myocardial infarction; CR, cardiac rupture; NST, no ST-segment elevation myocardial infarction; VSR, ventricular septal rupture; PMR, papillary muscle rupture; FWR, free wall rupture; WBC, white blood cell; PCI, percutaneous coronary intervention; CABG, coronary artery bypass grafting.

### 3.2 Sex difference in AMI patients with cardiac rupture

The clinical CR data were analyzed in terms of gender; the results are shown in Supplementary Table 3. Compared with male CR patients, female CR patients had a higher TG level and prevalence of diabetes (*p* < 0.05) but lower levels of Cr and spent a shorter time in-hospital (*p* < 0.05).

### 3.3 Age difference in AMI patients with cardiac rupture

The clinical CR data were analyzed in terms of age; the results are shown in Supplementary Table 4. More than 50% of female patients were more than 65 years old. CK-MB, high-density lipoprotein (HDL), very low-density lipoprotein (VLDL), RBC counts, Hb level, in-hospital mortality, the prevalence of diabetes, and time spent in hospital were statistically significant (*p* < 0.05). Next, we carried out a stratification analysis based on gender to assess the difference between the more than 65 years and under 65 years groups. Compared with male CR patients under 65 years of age, male CR patients more than 65 years of age had lower CK-MB, CK, HDL, VLDL, LDH, and hydroxybutyrate dehydrogenase (HBDH) levels (*p* < 0.05). Furthermore, male CR patients more than 65 years of age had a shorter in-hospital time and time of admission (*p* < 0.05) (see Supplementary Table 5). However, we did not find significant differences in female CR patients.

### 3.4 Risk factors for CR

**Figure 3:**
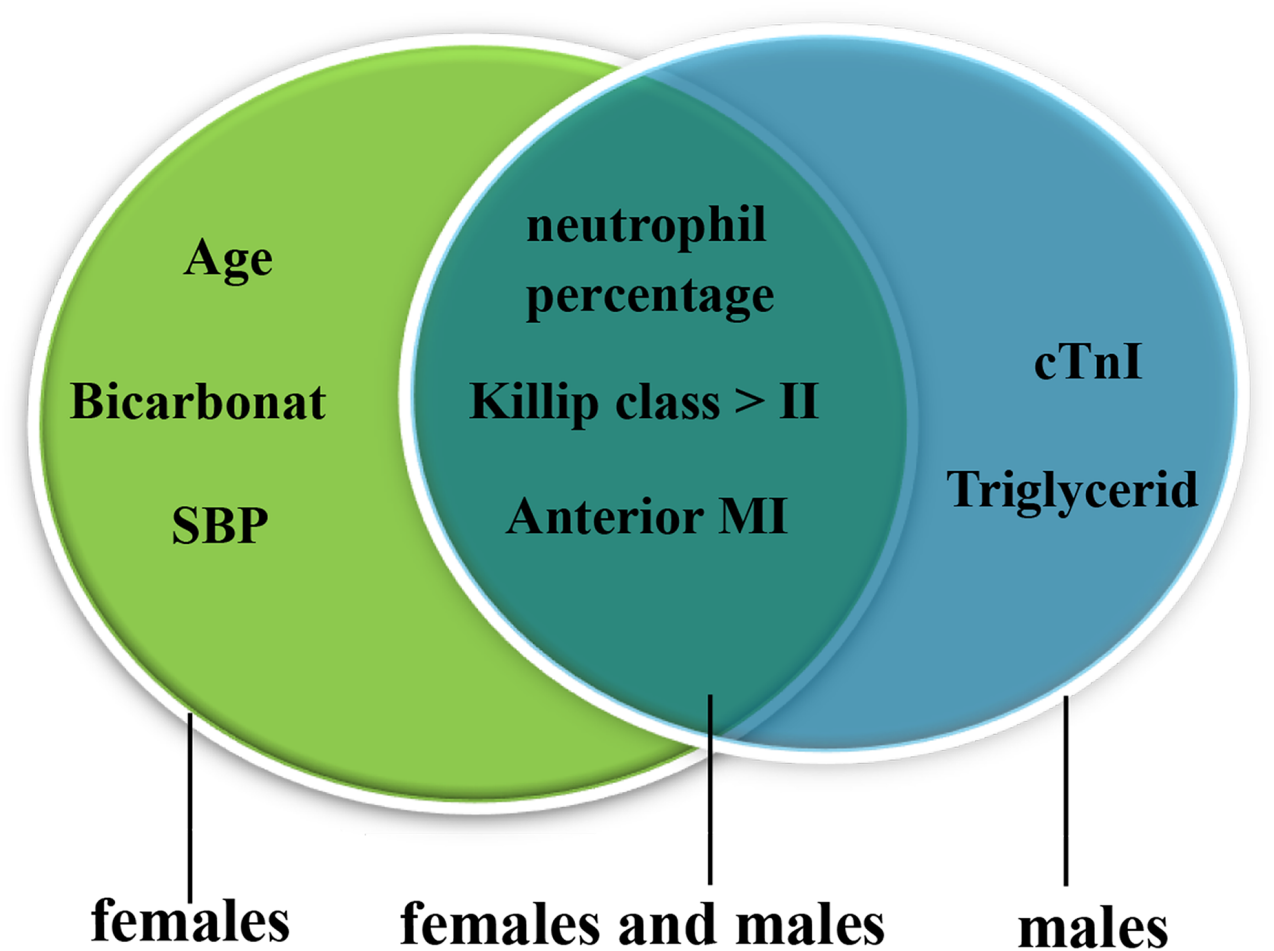
Risk factors for male and female CR patients. The green oval represents the risk factors for females, and the blue oval represents the risk factors for males; the overlap represents the risk factors for females and males. CR, cardiac rupture; MI, myocardial infarction; SBP, systolic blood pressure; cTnI, cardiac troponin I.

Univariate logistic regression identified that the cTnI level, WBC counts, the neut%, the Cr level, anterior MI, a Killip class of >II, SBP, LVEF, the albumin level, and the TG level were associated with CR (*p* < 0.05). The results after adjusting for age and sex were the same (Supplementary Table 6). Multivariate logistic regression discovered that high WBC counts [odds ratio (OR) = 1.258, 95% confidence interval (CI) 1.104–1.443, *p* = 0.001], a high neut% (OR = 1.063, 95% CI 1.004–1.125, *p* = 0.036), anterior MI (OR = 8.406, 95% CI 3.036–23.273, *p* < 0.001), and a Killip class of >II (OR = 4.517, 95% CI 1.399–14.582, *p* = 0.012) were risk factors for CR. In contrast, high albumin (OR = 0.869, 95% CI 0.773–0.978, *p* = 0.020) was protective against CR (shown in Table 3). We further examined the risk factors with CR in different genders. When conducting stratifying analysis by gender, we found that age (OR = 1.096, 95% CI 1.002–1.197, *p* = 0.044), SBP (OR = 0.936, 95% CI 0.899–0.975, *p* = 0.002), and bicarbonate (OR = 0.775, 95% CI 0.612–0.983, *p* = 0.035) were independently associated with female CR patients but not with male patients. The cTnI level (OR = 1.051, 95% CI 1.023–1.079, *p* < 0.001) and TG level (OR = 0.169, 95% CI 0.032–0.896, *p* = 0.037) were independently associated with male CR patients but not with female CR patients. Neut% (female, OR = 1.139, 95% CI 1.029–1.260, *p* = 0.012; male, OR = 1.108, 95% CI 1.025–1.199, *p* = 0.010), anterior MI (female, OR = 5.977, 95% CI 1.025–34.838, *p* = 0.047; male, OR = 11.761, 95%CI 2.651–52.177, *p* = 0.001), and a Killip class of >II (female, OR = 13.400, 95% CI 1.146–156.652, *p* = 0.039; male, OR = 11.901, 95% CI 2.273–62.314, *p* = 0.003) were independently associated with CR between females and males (see Figure 3, see more details in Supplementary Table 7).

**Table 3:** Multivariate logistic regression analysis for CR.

Variables	*β*	OR (95% CI)	*p*-value
Sex	0.479	1.614 (0.613–4.251)	0.332
Age	0.025	1.025 (0.977–1.075)	0.312
SBP	−0.006	0.994 (0.976–1.013)	0.556
BMI	−0.050	0.951 (0.830–1.090)	0.469
LVEF	0.010	1.010 (0.955–1.069)	0.722
cTnI	0.007	1.007 (0.996–1.017)	0.208
LDH	−0.002	0.998 (0.994–1.002)	0.364
HBDH	0.001	1.001 (0.997–1.005)	0.707
WBC	0.229	1.258 (1.104–1.443)	0.001**
Neut%	0.061	1.063 (1.004–1.125)	0.036*
Albumin	−0.140	0.869 (0.773–0.978)	0.020*
Creatinine	0.001	1.001 (0.993–1.008)	0.863
Triglyceride	−0.812	0.444 (0.161–1.225)	0.117
Bicarbonate	−0.070	0.932 (0.829–1.049)	0.246
MI location (anterior MI)	2.129	8.406 (3.036–23.273)	<0.001**
Killip class (>II)	1.508	4.517 (1.399–14.582)	0.012*

CR, cardiac rupture; MI, myocardial infarction; BMI, body mass index; SBP, systolic blood pressure; LVEF, left ventricular ejection fraction; cTnI, cardiac troponin I; LDH, lactate dehydrogenase; HBDH, hydroxybutyrate dehydrogenase; WBC, white blood cell; Neut%, neutrophil percentage; NST, no ST-segment elevation myocardial infarction, OR, odds ratio; CI, confidence interval.

* *p* < 0.05; **p ≤ 0.001.

### 3.5 Factors for in-hospital mortality

**Figure 4:**
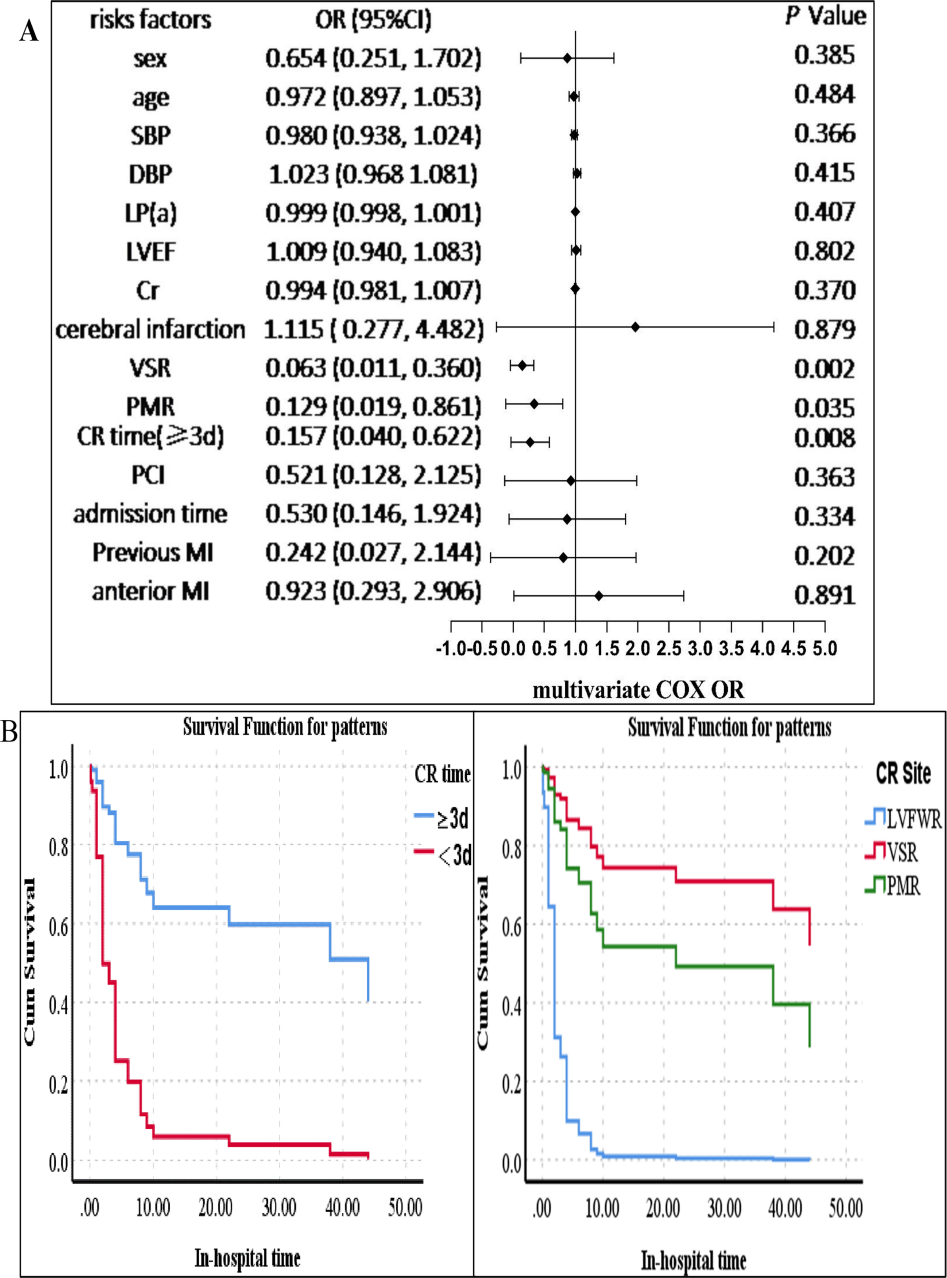
Survival analysis of the CR patients. (**A**) Multivariate Cox analysis of factors associated with in-hospital mortality. (**B**) Left: the survival probability of the patients in hospital. The survival probability of patients with a longer CR time (≥3 days) was higher than that of those with a shorter CR time (<3 days). The blue line indicates the group of patients with a CR time of ≥3 days, and the red line represents the group of patients with a CR time of <3 days. Right: the survival probability of patients with VSR or PMR was higher than that of those with FWR. The blue line indicates the group of patients with FWR, the red line represents the group of patients with VSR, and the green line indicates the group of patients with PMR. The *p*-value was estimated using multivariate Cox regression. SBP, systolic blood pressure; DBP, diastolic blood pressure; VSR, ventricular septal rupture; PMR, papillary muscle rupture; FWR, free wall rupture; PCI, percutaneous coronary intervention; cerebral infarction, previous cerebral infarction; CI, confidence interval; MI, myocardial infarction; CR, cardiac rupture; LVEF, left ventricular ejection fraction; Cr, creatinine; LP(a), lipoprotein(a). CR time, the time from symptom to CR (≥3 and <3 days); admission time, the time from symptom to admission (≥1 and <1 day).

Univariate and multivariate Cox regression analysis was used to estimate the OR and 95% CIs for in-hospital mortality. Univariate analysis revealed that age, an elevated lipoprotein(a) level, and previous cerebral infarction were related to a high in-hospital mortality risk (*p* < 0.05). Nevertheless, the site of CR (VSR) and a longer CR time (the time from symptom to CR) were connected with a low in-hospital mortality risk (*p* < 0.05) (see Supplementary Table 8 for details). In multivariate analysis, predictors of in-hospital mortality included the CR time (≥3 days) (OR = 0.157, 95% CI 0.040–0.622, *p* = 0.008), the site of CR [PMR (OR = 0.129, 95% CI 0.019–0.861, *p* = 0.035) and VSR (OR = 0.063, 95% CI 0.011–0.360, *p* = 0.002)]. The results are shown in Figure 4. We established that, in addition to CR time (*p* < 0.05) and the site of CR (*p* < 0.05), higher platelet counts (*p* < 0.05) were associated with a low in-hospital mortality for male CR patients according to stratification analysis, which was the same in female CR patients (see Table 4).

**Table 4:** Multivariate Cox regression analysis, stratified by gender for CR.

Variables	Female			Male		
	HR	95% CI	*p*-value	HR	95% CI	*p*-value
Age	0.971	0.868–1.086	0.602	0.962	0.868–1.066	0.458
BMI	0.896	0.661–1.215	0.481	1.069	0.843–1.356	0.583
SBP	1.002	0.969–1.035	0.916	0.990	0.963–1.018	0.496
PLT	0.986	0.973–0.999	0.038*	1.012	1.002–1.022	0.018*
CR time	0.152	0.025–0.923	0.041*	0.216	0.058–0.808	0.023*
Admission time	1.652	0.332–8.211	0.539	3.202	0.562–18.241	0.190
Site of CR			0.008*			0.023*
VSR	0.017	0.001–0.222	0.002*	0.077	0.012–0.479	0.006*
PMR	0.769	0.040–14.608	0.861	0.352	0.055–2.240	0.269

CR, cardiac rupture; BMI, body mass index; SBP, systolic blood pressure; PLT, platelets; VSR, ventricular septal rupture; PMR, papillary muscle rupture; HR, Hazard ratio; CI, confidence interval; CR time, the time from symptom to CR; admission time (≥3 and <3 days), the time from symptom to admission (≥1 and <1 day).

* *p* < 0.05.

## 4 Discussion

For cases with AMI, CR remains a mechanical complication with challenges in every aspect, from diagnosis to treatment. Different sites and the extent and types of CR lead to different prognostic outcomes (9). The incidence of CR was different in our group than in previous studies due to the difficulty of diagnosis in or out of the hospital. Recurrent chest pain, hypotension, and sudden death are the main clinical symptoms of CR. The new emergence of systolic murmurs may prompt VSR or PMR, and sudden cardiac death or electromechanical dissociation may indicate acute FWR (10, 11). In the present study, we found that WBC counts, neutrophil percentage, the albumin level, anterior AMI, and a Killip class of >II were independently associated with the development of CR.

Previous studies have indicated that the cardiac inflammatory response is a primary mechanism of heart rupture. The systematic inflammatory response could be activated after AMI, accompanying a mass of inflammatory cell infiltration in the heart. This is characterized by the infiltration of monocytes, neutrophils, and macrophages. Nevertheless, an excessive inflammatory response may result in post-infarct CR (5). Matrix metalloproteinase 9 (MMP-9) of infarct zones is derived from inflammatory cells that infiltrate the infarct myocardium. Activated MMP-9 could degrade the complete extracellular matrix. An excessive inflammatory response increases MMP-9 activity, which can result in defective myocardial remodeling and even the rupture of the myocardium (5). Inflammatory markers abnormally increased probably because inflammatory cell infiltration and MMP-9 expression were significantly higher in cardiac rupture (12). However, the mechanism still needs to be further studied. At present, some clinical studies have already indicated that neutrophil and WBC counts are associated with CR (13, 14). Similar outcomes were discovered in our present study. The anterior wall, primary AMI cases, and single-vessel disease, especially left anterior descending branch (LAD) involvement, are more likely to occur in CR, probably due to insufficient collateral circulation and ischemic preconditioning of the myocardium related to heat shock proteins (15, 16). In general, higher Killip classification may indicate larger infarct size. Research in the mouse MI model demonstrated that the reduction in infarcted muscle tensile strength depends on infarct size. Infarct size is the prerequisite for the rupture event (17). Our cohort confirmed these previously reported findings.

Furthermore, the blunted fibrotic healing constitutes another pathogenesis of cardiac rupture (5). Serum albumin, a widely accepted indicator of nutritional status, indicates protein-energy malnutrition resulting from the stress of illness, injury, or infection (18). In addition, hypoalbuminemia is also a sign of systemic inflammation. Research suggests that albumin levels are an independent predictor of new-onset heart failure and in-hospital mortality in patients with acute coronary syndromes (ACS) (19). Our study revealed that albumin is an independent predictor of CR after AMI. On the one hand, the decrease in the plasma albumin level may affect the ability of myocardial fiber regeneration and repair (20, 21). On the other hand, hypoalbuminemia may be a sign of heart inflammation. In the stratification analysis, there was no significant difference in albumin. This may be attributed to the affection of other variables or a relatively small number of CRs. The differences in the myocardial collagen matrix, differences in the inflammatory response, less well-developed collaterals, genetic differences, lower heart fat, and weaker myocardial fibers all contribute to the significantly higher proportion of females in MC patients than males (22, 23). Although it has also been reported that factors such as age, blood pressure, and positive initial cardiac biomarkers are independently related to a higher rate of CR (24), the gender difference in risk factors for CR has not been reported. In our study, we found there was a gender difference in the risk factors of CR. The reason for this difference in distribution is unclear.

In our patient cohort, the incidence of CR was 3.0% and the mortality rates were approximately 57%, which were similar to a previous study (23) and suggested lower morbidity and higher mortality. The mortality may be higher due to some patients abandoning treatment with an automatic discharge. Female sex has been reported to be associated with higher in-hospital mortality and be a predictor for MC following AMI (23). However, in this study, gender was not a predictor for in-hospital mortality. This is perhaps partly because of better economic conditions, the increased awareness of AMI, timely reperfusion therapy and revascularization, a limited sample size, and a high proportion of older women. Sex disparity was prominent in females under 60 years of age, with older age associated with a less significant gap in mortality (23). Furthermore, we discovered that a shorter CR time (<3 days) and FWR are independent risk factors for in-hospital mortality. FWR is the most dangerous, even highly fatal MC of AMI, which leads to pericardial tamponade due to bleeding into the pericardial cavity ultimately (4). The research found that the in-hospital mortality of FWR was up to 92.3% due to the sudden onset, rapid progression, and difficulty in treatment (25). Moreover, previous studies have also indicated that a longer AMI-FWR time is associated with lower in-hospital mortality in patients with FWR (26). This may be related to the myocardium being weak within the 3 days after AMI (5, 27). Although the results of subgroup analyses by gender were consistent, it does not mean there are no gender differences in risk factors, as other factors may have affected this result.

The early use of drugs such as β-blockers with ACEI/ARB (Angiotensin Converting Enzyme Inhibitors/Angiotensin Receptor Blockers) and primary PCI therapy may reduce the risk of CR after AMI (7). Surgical repair is the most effective treatment for CR (28). Unfortunately, in one study, more than 90% of cases waiting for surgery died with conservative management (29). The balance of extracellular fluid volume is significantly important. Surgery can be delayed for 3–4 weeks in patients with hemodynamic stability. If the condition worsens, emergency surgery should be performed (30). However, the sudden circulatory collapse and a delay in the initial response might have made it difficult to perform emergency surgery, and mortality after surgical treatment is also high. Hence, it is very important to identify risk factors associated with CR early.

## 5 Limitations

The study had the following limitations: (1) this study was a single-center small-sample clinical retrospective study. The system biases may have influenced our analyses. In addition, the sample size was limited, particularly for subgroup analysis. Moreover, our results may have been affected by confounders not included in this study. (2) Given that all of our research subjects were Chinese patients with CR, the generalizability of our findings to other patient populations may be limited.

## 6 Conclusions

A low albumin level, high WBC counts, anterior myocardial infarction, and a Killip class of >II were independent predictors for CR. However, risk factors associated with CR are different in male and female patients. The time from symptom to CR, the site of CR, and platelet counts were independent predictors for in-hospital mortality from CR. These may be helpful in the early and accurate identification of CR and the assessment of prognosis. Gender differences should be considered.

## Data Availability

The original contributions presented in the study are included in the article/Supplementary Material, further inquiries can be directed to the corresponding authors.
