# Supplementary material for: Factors related to cardiac rupture after acute myocardial infarction
Source: Front Cardiovasc Med. 2024 Oct 2;11:1401609. doi: 10.3389/fcvm.2024.1401609 (PMC11479954; doi:10.3389/fcvm.2024.1401609)
Supplement: Supplementary file 1 [file Datasheet1.zip › Supplementary Material/Table 1 .docx]

Table 1. The clinical characteristics of AMI patients with or without CR.

| **Variables** | **No-CR (n=1648)** | **CR (n=51)** | ***P*-value** |
| --- | --- | --- | --- |
| Age (*year*) | 63.84 ± 11.64 | 70.47 ± 8.88 | <0.001** |
| Female (*n, %*) | 485 (29%) | 22/51 (43%) | 0.035* |
| Hypertension (*n, %*) | 756 (46%) | 27 (53%) | 0.319 |
| Cerebral infarction(*n, %*) | 213 (13%) | 7 (14%) | 0.086 |
| Previous MI (*n, %*) | 136 (8%) | 3 (6%) | 0.727 |
| DM (*n, %*) | 387 (23%) | 13 (25%) | 0.994 |
| BMI (*kg/m2*) | 24.78 ± 3.55 | 23.50 ± 3.10 | 0.010* |
| SBP (*mmHg*) | 124.96 ± 23.77 | 113.25 ± 26.05 | 0.001** |
| DBP (*mmHg*) | 76.84 ± 15.29 | 73.88 ± 19.13 | 0.067 |
| LVEF (*%*) | 48.63 ± 7.84 | 44.96 ± 7.74 | 0.010* |
| cTnI (*ng/ml*) | 2.24 (0.22-11.64) | 17.51 (5.25-48.19) | <0.001** |
| MYO (*ng/ml*) | 107.40 (40.82-325.68) | 236.50 (80.90-540.38) | 0.002* |
| CK (*U/L*) | 906.00 (280.00-1928.25) | 609.00 (247.00-1536.00) | 0.228 |
| CK-MB (*ng/ml*) | 74.00 (28.00-168.00) | 58.00 (15.00-151.20) | 0.179 |
| LDH (*U/L*) | 480.00 (300.00-763.00) | 555.00 (387.00-907.00) | 0.024 |
| HBDH (*U/L*) | 458.00 (268.00-763.25) | 515.00 (324.00-839.00) | 0.107 |
| Glucose (*mmol/L*) | 6.10 (5.00-8.20) | 7.30 (5.80-9.60) | 0.003* |
| WBC (*×10^9/L*) | 9.71 (7.70-12.00) | 13.02 (10.98-16.01) | <0.001** |
| RBC (*×10^12/L*) | 4.27 ± 0.60 | 4.16 ± 0.65 | 0.030* |
| Hb (*g/L*) | 130.83 ± 18.69 | 127.92 ± 19.61 | 0.014* |
| PLT (*×10^9/L*) | 221.00 (185.00-260.00) | 233.00 (180.00-281.00) | 0.324 |
| Neut% (*%*) | 76.50 (69.60-82.70) | 85.10 (75.30-88.00) | <0.001** |
| TP (*g/L*) | 62.52 ± 5.85 | 61.34 ± 5.92 | 0.097 |
| Albumin (*g/L*) | 38.04 ± 3.84 | 35.81 ± 4.80 | <0.001** |
| Cr (*µmol/L*) | 65.80 (56.20-77.18) | 76.60 (61.00-104.70) | 0.001** |
| HDL (*mmol/L*) | 1.08 ± 0.34 | 1.10 ± 0.31 | 0.444 |
| LDL (*mmol/L*) | 2.59 ± 0.75 | 2.62 ± 0.79 | 0.904 |
| VLDL (*mmol/L*) | 0.60 ± 0.40 | 0.47 ± 0.24 | 0.043* |
| Lipoprotein(a) (*mg/L*) | 198.00 (99.00-351.00) | 214.00 (123.00-403.00) | 0.291 |
| TG (*mmol/L*) | 1.22 (0.88-1.73) | 0.95 (0.73-1.25) | <0.001** |
| TC (*mmol/L*) | 4.25 ± 0.95 | 4.17 ± 0.95 | 0.301 |
| Bicarbonate (*mmol/L*) | 24.11 ± 3.54 | 22.78 ± 4.62 | 0.098 |
| Site of AMI (*n, %*) |  |  | <0.001** |
| anterior | 610 (37%) | 35 (69%) |  |
| No-anterior | 1038 (63%) | 16 (31%) |  |
| Killip class (*n, %*) |  |  | <0.001** |
| ≤II | 1518 (92%) | 32 (62%) |  |
| >II | 130 (8%) | 19 (38%) |  |

AMI, acute myocardial infarction; CR, cardiac rupture; cerebral infarction, previous cerebral infarction; MI, myocardial infarction; BMI, body mass index; DBP, diastolic blood pressure; SBP, systolic blood pressure; DM, diabetes mellitus; LVEF, left ventricular ejection fraction; cTnI, cardiac troponin I; MYO, myoglobin; CK, creatine kinase; CK-MB, creatine kinase isoenzymes B; LDH, lactate dehydrogenase; HBDH, hydroxybutyrate dehydrogenase; WBC, white blood cell; RBC, red blood cell; Hb, hemoglobin; PLT, Platelets; Neut%, neutrophil percentage; TP, total protein; Cr, creatinine; HDL, high density lipoprotein; LDL, Low Density Lipoprotein; VLDL, very low-density lipoprotein; TG, Triglyceride; TC, total cholesterol. **P* < 0.05; ***P* **≤** 0.001.
