# Supplementary material for: Factors related to cardiac rupture after acute myocardial infarction
Source: Front Cardiovasc Med. 2024 Oct 2;11:1401609. doi: 10.3389/fcvm.2024.1401609 (PMC11479954; doi:10.3389/fcvm.2024.1401609)
Supplement: Supplementary file 1 [file Datasheet1.zip › Supplementary Material/Table 2.docx]

Table 2. The clinical characteristics of CR patients, stratified by gender.

| **Variables** | | **Female (n=507)** | | | | ***P*-value** | |
| --- | --- | --- | --- | --- | --- | --- | --- |
|  |  | **No-CR(n=485)** | | **CR(n=22)** | |  |  |
| Age (*year*) | | 70 (63,77) | | 72.59±8.50 | | 0.239 | |
| Hypertension (*n, %*) | |  | |  | | 0.477 | |
| No | | 227 (46.8%) | | 12(54.5%) | |  | |
| Yes | | 258 (53.2%) | | 10(45.5%) | | 1.000 | |
| Cerebral infarction (*n, %*) | |  | |  | |  | |
| No | | 422(87%) | | 19(86.4) | |  | |
| Yes | | 63(13%) | | 3(13.6) | |  | |
| Previous MI (*n, %*) | |  | |  | | 1.000 | |
| No | | 455(93.8%) | | 21(95.5%) | |  | |
| Yes | | 30(6.2%) | | 1(4.5%) | |  | |
| DM (*n, %*) | |  | |  | | 0.416 | |
| No | | 327(67.4%) | | 13(59.1) | |  | |
| Yes | | 158(32.6%) | | 9(40.1) | |  | |
| BMI (*kg/m2*) | | 24.28±3.71 | | 23.47±3.27 | | 0.319 | |
| SBP (*mmHg*) | | 127.13±25.00 | | 106.73±23.40 | | <0.001** | |
| DBP (*mmHg*) | | 75.00(67, 86) | | 64.50(55.75, 79.00) | | 0.010* | |
| LVEF (*%*) | | 50.00(43, 54) | | 45.00(39.75, 52.00) | | 0.143 | |
| cTnI (*ng/ml*) | | 2.06(0.39, 10.03) | | 14.45(6.11, 48.43) | | <0.001** | |
| MYO (*ng/ml*) | | 113.10(38.95, 321.55) | | 277.81(78.47, 550.29) | | 0.026* | |
| CK (*U/L*) | | 704.00(219.00,1535.50) | | 814.00(202.25, 1404.50) | | 0.847 | |
| CK-MB (*ng/ml*) | | 61.00(24.65, 150.72) | | 61.00(10.75, 152.93) | | 0.543 | |
| LDH (*U/L*) | | 468.00(300.00, 716.50) | | 523.00(409.75, 804.25) | | 0.189 | |
| HBDH (*U/L*) | | 444.00(265.00, 708.00) | | 508.50(351.50, 794.00) | | 0.232 | |
| Glucose (*mmol/L*) | | 6.50(5.20, 9.60) | | 8.92±3.22 | | 0.029* | |
| WBC (*×10^9/L*) | | 9.60(7.21, 11.90) | | 12.42(10.94, 16.83) | | <0.001** | |
| RBC (*×10^12/L*) | | 4.07±0.57 | | 4.07±0.62 | | 0.974 | |
| Hb (*g/L*) | | 121.54±16.15 | | 124.73±17.69 | | 0.368 | |
| PLT (*×10^9/L*) | | 238.00(197.00, 277.50) | | 265.50(220.25, 318.75) | | 0.161 | |
| Neut% (*%*) | | 77.40(69.50, 83.60) | | 82.43(73.97, 87.70) | | 0.050* | |
| TP (*g/L*) | | 63.45±6.17 | | 60.73±6.51 | | 0.044* | |
| Albumin (*g/L*) | | 37.68±4.03 | | 35.72±5.16 | | 0.028* | |
| Cr (*µmol/L*) | | 56.70(48.20, 68.45) | | 61.70(51.28, 83.73) | | 0.089 | |
| HDL (*mmol/L*) | | 1.13(0.96, 1.29) | | 1.08±0.24 | | 0.364 | |
| LDL (*mmol/L*) | | 2.71(2.20, 3.30) | | 2.60(2.20,3.60) | | 0.992 | |
| VLDL (*mmol/L*) | | 0.56(0.37, 0.84) | | 0.47(0.31, 0.71) | | 0.099 | |
| Lipoprotein(a) (*mg/L*) | | 228.00(116.00, 397.50) | | 266.50(126.75, 502.75) | | 0.562 | |
| TG (*mmol/L*) | | 1.27(0.97, 1.83) | | 1.09(0.93,1.50) | | 0.126 | |
| TC (*mmol/L*) | | 4.48(3.90, 5.23) | | 4.41±1.01 | | 0.297 | |
| Bicarbonate (*mmol/L*) | | 23.80(21.30, 26.05) | | 22.17±4.18 | | 0.165 | |
| Site of AMI (*n, %*) | |  | |  | | 0.001** | |
| anterior | | 164(66.2%) | | 15(68.2%) | |  | |
| No-anterior | | 321(33.8%) | | 7(31.8%) | |  | |
| Killip class (*n, %*) | |  | |  | | <0.001** | |
| ≤II | | 439 (90.5%) | | 14 (63.6%) | |  | |
| >II | | 46 (9.5%) | | 8 (36.4%) | |  | |
| **Variables** | | **Male (n=1192)** | | | | ***P*-value** | |
|  |  | **No-CR(n=1163)** | | **CR(n=29)** | |  |  |
| Age (*year*) | | 62 (53, 69) | | 70 (64, 76) | | <0.001** | |
| Hypertension (*n, %*) | |  | |  | | 0.090 | |
| No | | 665 (57.2%) | | 12 (41.4%) | |  | |
| Yes | | 498 (42.8%) | | 17 (58.6%) | |  | |
| Cerebral infarction (*n, %*) | |  | |  | | 1.000 | |
| No | | 1013 (87.1%) | | 25 (86.2%) | |  | |
| Yes | | 150 (12.9%) | | 4 (13.8%) | |  | |
| Previous MI (*n, %*) | |  | |  | | 0.933 | |
| No | | 1057 (90.9%) | | 27 (93.1%) | |  | |
| Yes | | 106 (9.1%) | | 2 (6.9%) | |  | |
| DM (*n, %*) | |  | |  | | 0.429 | |
| No | | 934(80.3%) | | 25(86.2%) | |  | |
| Yes | | 229(19.7%) | | 4(13.8%) | |  | |
| BMI (*kg/m2*) | | 24.80(22.50, 27.04) | | 23.37(20.95, 25.11) | | 0.023* | |
| SBP (*mmHg*) | | 123.00(108.00, 138.00) | | 118.21±27.24 | | 0.265 | |
| DBP (*mmHg*) | | 77.00(68, 87) | | 76.90±17.76 | | 0.907 | |
| LVEF (*%*) | | 50.00(44, 54) | | 46.00(38.50, 50.00) | | 0.002* | |
| cTnI (*ng/ml*) | | 2.31(0.17, 12.37) | | 17.68(3.28, 49.72) | | <0.001** | |
| MYO (*ng/ml*) | | 105.08(41.90, 330.06) | | 225.66(79.19, 499.01) | | 0.024* | |
| CK (*U/L*) | | 983.00(321.00,2087.00) | | 600.00(249.45, 1609.50) | | 0.266 | |
| CK-MB (*ng/ml*) | | 81.00(30.00, 175.00) | | 55.00(17.00, 153.62) | | 0.300 | |
| LDH (*U/L*) | | 484.00(300.00, 778.00) | | 735.00(366.00, 1036.00) | | 0.052 | |
| HBDH (*U/L*) | | 465.00(269.00, 773.00) | | 616.00(275.50, 939.00) | | 0.208 | |
| Glucose (*mmol/L*) | | 5.90(4.90, 7.80) | | 6.50(5.50, 8.40) | | 0.086 | |
| WBC (*×10^9/L*) | | 9.80(7.72, 12.03) | | 13.38(10.90, 15.76) | | <0.001** | |
| RBC (*×10^12/L*) | | 4.52(4.18, 4.84) | | 4.34(3.60, 4.65) | | 0.024* | |
| Hb (*g/L*) | | 140.00(129.00, 150.00) | | 129.00(113.00, 147.00) | | 0.008* | |
| PLT (*×10^9/L*) | | 215.00(181.00, 254.00) | | 220.00(167.00, 260.50) | | 0.835 | |
| Neut% (*%*) | | 76.20(69.60, 82.34) | | 85.74(77.00, 88.41) | | <0.001** | |
| TP (*g/L*) | | 61.12±5.54 | | 61.81±5.51 | | 0.765 | |
| Albumin (*g/L*) | | 38.64±3.85 | | 35.89±4.60 | | <0.001** | |
| Cr (*µmol/L*) | | 68.20(60.30, 79.00) | | 83.80(69.60, 118.40) | | <0.001** | |
| HDL (*mmol/L*) | | 1.02(0.88, 1.19) | | 1.12±0.35 | | 0.228 | |
| LDL (*mmol/L*) | | 2.48(2.02, 3.00) | | 2.45±0.67 | | 0.623 | |
| VLDL (*mmol/L*) | | 0.50(0.32, 0.74) | | 0.45±0.24 | | 0.128 | |
| Lipoprotein(a) (*mg/L*) | | 189.00(95.00, 334.00) | | 187.00(121.00, 391.00) | | 0.522 | |
| TG (*mmol/L*) | | 1.20(0.85, 1.69) | | 0.81(0.62,1.07) | | <0.001** | |
| TC (*mmol/L*) | | 4.13(3.49, 4.81) | | 3.98±0.87 | | 0.348 | |
| Bicarbonate (*mmol/L*) | | 23.60(21.60, 26.00) | | 23.25±4.95 | | 0.294 | |
| Site of AMI (*n, %*) | |  | |  | | 0.001** | |
| anterior | | 446 (38.3%) | | 20 (69.0%) | |  |  |
| No-anterior | | 717 (61.7%) | | 9 (31.0%) | |  |  |
| Killip class (*n, %*) | |  | |  | | <0.001** | |
| ≤II | | 1079 (93.8%) | | 18(62.1%) | |  | |
| >II | | 84 (7.2) | | 11(37.9%) | |  | |

AMI, acute myocardial infarction; CR, cardiac rupture; cerebral infarction, previous cerebral infarction; MI, myocardial infarction; BMI, body mass index; DBP, diastolic blood pressure; SBP, systolic blood pressure; DM, diabetes mellitus; LVEF, left ventricular ejection fraction; cTnI, cardiac troponin I; MYO, myoglobin; CK, creatine kinase; CK-MB, creatine kinase isoenzymes B; LDH, lactate dehydrogenase; HBDH, hydroxybutyrate dehydrogenase; WBC, white blood cell; RBC, red blood cell; Hb, hemoglobin; PLT, Platelets; Neut%, neutrophil percentage; TP, total protein; Cr, creatinine; HDL, high density lipoprotein; LDL, Low Density Lipoprotein; VLDL, very low-density lipoprotein; TG, Triglyceride; TC, total cholesterol. **P* < 0.05; ***P* **≤** 0.001.
