# Supplementary material for: Factors related to cardiac rupture after acute myocardial infarction
Source: Front Cardiovasc Med. 2024 Oct 2;11:1401609. doi: 10.3389/fcvm.2024.1401609 (PMC11479954; doi:10.3389/fcvm.2024.1401609)
Supplement: Supplementary file 1 [file Datasheet1.zip › Supplementary Material/Table 3.docx]

Table 3. Sex difference in AMI patients with CR

| Variables | Male (29) | Female (22) | *P*-value |
| --- | --- | --- | --- |
| Age (*years*) | 68.86 ± 8.97 | 72.59 ± 8.50 | 0.139 |
| Admission time (*days*) | 2.00 (0.53-6.50) | 1.04 (0.29-4.25) | 0.321 |
| CR time (*days*) | 5.00 (2.04-9.04) | 3.17 (1.24-7.00) | 0.116 |
| In-hospital time (*days*) | 6.00 (2.00-30.00) | 2.50 (1.00-5.25) | 0.022* |
| In-hospital death (*n, %*) | 15 (52%) | 14 (64%) | 0.395 |
| PCI (*n, %*) | 9 (31%) | 4 (18%) | 0.297 |
| Sit of CR (*n, %*) |  |  | 0.499 |
| FWR | 9 (31%) | 10 (45%) |  |
| VSR | 15 (52%) | 10 (45%) |  |
| PMR | 5 (17%) | 2 (9%) |  |
| BMI (*kg/m^2^*) | 23.51 ± 3.02 | 23.47 ±3.27 | 0.960 |
| Sit of AMI (*n, %*) |  |  | 0.952 |
| anterior | 20 (69%) | 15 (68%) |  |
| no-anterior | 9 (31%) | 7 (32%) |  |
| DM (*n, %*) | 4 (14%) | 9 (41%) | 0.028* |
| Cerebral infarction (*n, %*) | 4 (14%) | 3 (14%) | 1.000 |
| Previous MI (*n, %*) | 2 (7%) | 1 (5%) | 1.000 |
| Hypertension (*n, %*) | 17 (59%) | 10 (45%) | 0.351 |
| SBP (*mmHg*) | 118.21 ± 27.24 | 106.73 ± 23.40 | 0.120 |
| DBP (*mmHg*) | 76.90 ± 17.76 | 69.91 ± 20.53 | 0.075 |
| LVEF (*%*) | 46.00 (38.50-50.00) | 45.00 (39.75-52) | 0.523 |
| HDL (*mmol/L*) | 1.13 (0.72-1.38) | 1.04 (0.92-1.32) | 0.621 |
| LDL (*mmol/L*) | 2.36 ± 0.69 | 2.85 ± 0.89 | 0.159 |
| VLDL (*mmol/L*) | 0.58 ± 0.61 | 0.50 ± 0.25 | 0.541 |
| Cr (*umol/L*) | 83.8 (69.60-123.95) | 61.70 (51.28-83.73) | 0.003* |
| WBC (**10^9/L*) | 13.38 (10.90-15.76) | 12.42 (10.94-16.83) | 0.530 |
| TG (*mmol/L*) | 0.81 (0.62-1.07) | 1.09 (0.93-1.50) | 0.005* |

AMI, acute myocardial infarction; CR, cardiac rupture; VSR, ventricular septal rupture; PWR, papillary muscle rupture; FWR, free wall rupture; PCI, percutaneous coronary intervention; BMI, body mass index; DM, diabetes mellitus; cerebral infarction, previous cerebral infarction; MI, myocardial infarction; DBP, diastolic blood pressure; SBP, systolic blood pressure; LVEF, left ventricular ejection fraction; HDL, high density lipoprotein; LDL, low density lipoprotein; VLDL, very low density lipoprotein; Cr, creatinine; WBC, white blood cell; TG, triglyceride. CR time, the time from Symptom to CR (≥3 days and <3 days); admission time, the time from Symptom to admission (≥1 day and <1 day). **P* < 0.05.
