# Supplementary material for: Factors related to cardiac rupture after acute myocardial infarction
Source: Front Cardiovasc Med. 2024 Oct 2;11:1401609. doi: 10.3389/fcvm.2024.1401609 (PMC11479954; doi:10.3389/fcvm.2024.1401609)
Supplement: Supplementary file 1 [file Datasheet1.zip › Supplementary Material/Table 4.docx]

Table 4. Age difference in AMI patients CR

| **Variables** | **Age>65 (35)** | **Age<=65 (16)** | ***P-*value** |
| --- | --- | --- | --- |
| Sex, Female (*n*, %) | 18 (51%) | 4 (25%) | 0.077 |
| Admission time (*days*) | 2.00 (0.33-7.00) | 1.50 (0.32-5.00) | 0.476 |
| CR time (*days*) | 3.92 (1.74-7.25) | 5.00 (2.19-7.22) | 0.633 |
| In-hospital time (*days*) | 2.00 (5.00-1.00) | 18.50 (3.75-43.25) | 0.003* |
| In-hospital death (*n, %*) | 20 (57%) | 9 (56%) | ≤0.001** |
| PCI (*n, %*) | 7 (20%) | 6 (38%) | 0.298 |
| Sit of CR (*n, %*) |  |  | 0.843 |
| FWR | 14 (40%) | 5 (31%) |  |
| VSR | 16 (46%) | 9 (56%) |  |
| PMR | 5 (14%) | 2 (13%) |  |
| Sit of AMI (*n, %*) |  |  | 0.524 |
| anterior | 25 (71%) | 10 (63%) |  |
| no-anterior | 10 (29%) | 6 (38%) |  |
| DM (*n, %*) | 4 (11%) | 9 (56%) | 0.028* |
| Cerebral infarction (*n, %*) | 5 (14%) | 2 (13%) | 1.000 |
| Previous MI (*n, %*) | 3 (9%) | 0 (0%) | 1.000 |
| Hypertension (*n, %*) | 17 (49%) | 10 (63%) | 0.351 |
| BMI (*kg/m^2^*) | 22.97 ± 2.98 | 24.66 ± 3.12 | 0.070 |
| SBP (*mmHg*) | 110.97 ± 25.64 | 118.25 ± 27.06 | 0.360 |
| DBP (*mmHg*) | 71.91 ± 19.31 | 78.19 ± 18.60 | 0.193 |
| LVEF (*%*) | 47.00 (40.00-50.00) | 43.00 (35.50-47.75) | 0.078 |
| CK (*U/L*) | 600.00 (220.00-1219.00) | 15111.30 (300.83-2121.13) | 0.074 |
| CK-MB (*ng/mL*) | 38.23 (11.00-118.00) | 113.00 (34.50-201.84) | 0.026* |
| LDH (*U/L*) | 492.00 (361.00-791.00) | 804.50 (451.50-1299.50) | 0.102 |
| HBDH (*U/L*) | 490.00 (269.00-823.00) | 794.00(823.00-1274.15) | 0.059 |
| HDL (*mmol/L*) | 1.01 ± 0.28 | 1.30 ± 0.27 | 0.001** |
| LDL (*mmol/L*) | 2.60 ± 0.73 | 2.67 ± 0.95 | 0.774 |
| VLDL (*mmol/L*) | 0.37 (0.27-0.50) | 0.58 (0.42-0.70) | 0.024* |
| WBC (**10^9/L)* | 12.35 (10.04-15.74) | 14.93 (12.80-16.40) | 0.071 |
| RBC *(*10^12/L)* | 4.03 ± 0.56 | 4.45 ± 0.75 | 0.029* |
| Hb (*g/L*) | 123.63 ± 15.44 | 137.31 ± 24.57 | 0.019** |

AMI, acute myocardial infarction; CR, cardiac rupture; VSR, ventricular septal rupture; PWR, papillary muscle rupture; FWR, free wall rupture; DM, diabetes mellitus; cerebral infarction, previous cerebral infarction; PCI, percutaneous coronary intervention; MI, myocardial infarction; BMI, body mass index; DBP, diastolic blood pressure; SBP, systolic blood pressure; LVEF, left ventricular ejection fraction; CK, creatine kinase; CK-MB, creatine kinase isoenzymes B; LDH, lactate dehydrogenase; HBDH, hydroxybutyrate dehydrogenase; HDL, high density lipoprotein; LDL, low density lipoprotein; VLDL, very low density lipoprotein; WBC, white blood cell; Hb, hemoglobin. CR time, the time from Symptom to CR (≥3 days and <3 days); admission time, the time from Symptom to admission (≥1 day and <1 day).
