# Supplementary material for: Factors related to cardiac rupture after acute myocardial infarction
Source: Front Cardiovasc Med. 2024 Oct 2;11:1401609. doi: 10.3389/fcvm.2024.1401609 (PMC11479954; doi:10.3389/fcvm.2024.1401609)
Supplement: Supplementary file 1 [file Datasheet1.zip › Supplementary Material/Table 5.docx]

Table 5. Age difference in CR patients, stratified by gender.

| **Variables** | **Female(n=22)** | | ***P-*value** |
| --- | --- | --- | --- |
|  | **Age>65 (n=18)** | **Age****≤65(n=4)** |  |
| Admission time (*days*) | 1.00(0.29, 3.25) | 3.75±1.89 | 0.135 |
| CR time (*days*) | 2.90(1.10, 5.50) | 5.99±2.68 | 0.115 |
| In-hospital time (*days*) | 2.00(1.00, 4.00) | 11±13.24 | 0.262 |
| In-hospital outcome (*n, %*) |  |  | 1.000 |
| death | 11 (38.9%) | 3 (75%) |  |
| survive | 7 (61.1%) | 1 (25%) |  |
| PCI (*n, %*) |  |  | 0.554 |
| No | 14 (77.8%) | 4 |  |
| Yes | 4 (22.2%) | 0 |  |
| Sit of CR (*n, %*) |  |  | 0.395 |
| FWR | 8 (44.4%) | 2 (50%) |  |
| VSR | 9 (50%) | 1 (25%) |  |
| PMR | 1 (5.6%) | 1 (25%) |  |
| Sit of AMI (*n, %*) |  |  | 0.077 |
| anterior | 14 (77.8%) | 1 (25.0%) |  |
| no-anterior | 4 (22.2%) | 3 (75.0%) |  |
| DM (*n, %*) |  |  | 1.000 |
| No | 11 (61.1%) | 2 (50%) |  |
| Yes | 7 (38.9%) | 2 (50%) |  |
| Cerebral infarction (*n, %*) |  |  | 1.000 |
| No | 16 (88.9%) | 3 (75.0%) |  |
| Yes | 2 (11.1%) | 1 (25.0%) |  |
| Previous MI (*n, %*) |  |  | 1.000 |
| No | 17 (94.4%) | 4 (100%) |  |
| Yes | 1 (5.6%) | 0 |  |
| Hypertension (*n, %*) |  |  | 1.000 |
| No | 10 (55.6%) | 2 (50%) |  |
| Yes | 8 (44.4%) | 2 (50%) |  |
| BMI (*kg/m^2^*) | 22.86±3.22 | 26.21±1.98 | 0.062 |
| SBP (*mmHg*) | 97.50(86.00, 123.50) | 105.00±20.61 | 0.932 |
| DBP (*mmHg*) | 64.00(55.75, 80.00) | 66.75±15.56 | 0.966 |
| LVEF (*%*) | 46.00(39.75, 52.25) | 44.25±5.74 | 0.638 |
| CK (*U/L*) | 814.00(215.00, 1254.75) | 917.28±918.82 | 0.865 |
| CK-MB (*ng/mL*) | 61.00(10.24, 137.22) | 86.79±79.23 | 0.798 |
| LDH (*U/L*) | 704.00±408.29 | 432.25±125.72 | 0.210 |
| HBDH (*U/L*) | 520.50(351.50, 910.75) | 428.10±135.35 | 0.287 |
| HDL (*mmol/L*) | 1.04±0.23 | 1.23±0.24 | 0.156 |
| LDL (*mmol/L*) | 2.79±0.79 | 3.13±1.38 | 0.509 |
| VLDL (*mmol/L*) | 0.48±0.27 | 0.55±0.16 | 0.626 |
| WBC (**10^9/L)* | 13.19±4.40 | 13.63±2.67 | 0.849 |
| RBC *(*10^12/L)* | 3.99±0.60 | 4.42±0.70 | 0.227 |
| Hb (*g/L*) | 122.78±16.96 | 133.50±20.87 | 0.284 |
| **Variables** | **Male** | | ***P-*value** |
|  | **Age>65(n=17)** | **Age≤65 (n=12)** |  |
| Admission time (*days*) | 3.00(1.50, 10.00) | 6.49±7.95 | 0.045* |
| CR time (*days*) | 6.00(2.04, 12.20) | 8.37±8.07 | 0.400 |
| In-hospital time (*days*) | 4.00(2.00, 7.50) | 8.77±12.84 | 0.019* |
| In-hospital outcome (*n, %*) |  |  | 1.000 |
| death | 9(52.9%) | 6(50%) |  |
| survive | 8(47.1%) | 6(50%) |  |
| PCI (*n, %*) |  |  | 0.106 |
| No | 14(82.4%) | 6(50%) |  |
| Yes | 3(17.6%) | 6(50%) |  |
| Sit of CR (*n, %*) |  |  | 0.409 |
| FWR | 6(35.3%) | 3(25%) |  |
| VSR | 7(41.2%) | 8(66.7%) |  |
| PMR | 4(23.5%) | 1(8.3%) |  |
| Sit of AMI (*n, %*) |  |  | 0.694 |
| anterior | 11(64.7%) | 9(75%) |  |
| no-anterior | 6(35.3%) | 3(25%) |  |
| DM (*n, %*) |  |  | 1.000 |
| No | 15(88.2%) | 10(83.3%) |  |
| Yes | 2(11.8%) | 2(16.7%) |  |
| Cerebral infarction (*n, %*) |  |  | 0.622 |
| No | 14(82.4%) | 11(91.7%) |  |
| Yes | 3(17.6%) | 1(8.3%) |  |
| Previous MI (*n, %*) |  |  | 0.498 |
| No | 15 (88.2%) | 12 |  |
| Yes | 2 (11.8%) | 0 |  |
| Hypertension (*n, %*) |  |  | 0.703 |
| No | 8(47.1%) | 4(33.3%) |  |
| Yes | 9(52.9%) | 8(66.7%) |  |
| BMI (*kg/m^2^*) | 23.07±2.81 | 24.14±3.32 | 0.359 |
| SBP (*mmHg*) | 115.06±26.92 | 122.67±28.24 | 0.469 |
| DBP (*mmHg*) | 73.29±16.83 | 82.00±18.50 | 0.199 |
| LVEF (*%*) | 46.06±5.64 | 40.67±9.15 | 0.088 |
| CK (*U/L*) | 430.00(187.50, 1088.70) | 1513.30(457.74, 2471.23) | 0.034* |
| CK-MB (*ng/mL*) | 28.00(11.10, 87.91) | 113.00(57.75, 353.25) | 0.008* |
| LDH (*U/L*) | 469.00(298.00, 743.50) | 976.58±433.84 | 0.007* |
| HBDH (*U/L*) | 330.00(257.00, 793.00) | 926.97±430.09 | 0.005* |
| HDL (*mmol/L*) | 0.98±0.33 | 1.32±0.29 | 0.008* |
| LDL (*mmol/L*) | 2.40±0.61 | 2.52±0.78 | 0.652 |
| VLDL (*mmol/L*) | 0.37±0.21 | 0.57±0.24 | 0.029* |
| WBC (**10^9/L)* | 12.46(9.97, 15.02) | 15.13±2.90 | 0.063 |
| RBC *(*10^12/L)* | 4.07±0.53 | 4.46±0.79 | 0.119 |
| Hb (*g/L*) | 124.53±14.12 | 138.58±26.41 | 0.113 |

AMI, acute myocardial infarction; CR, cardiac rupture; VSR, ventricular septal rupture; PWR, papillary muscle rupture; FWR, free wall rupture; DM, diabetes mellitus; Cerebral infarction, pervious cerebral infarction; PCI, percutaneous coronary intervention; MI, myocardial infarction; BMI, body mass index; DBP, diastolic blood pressure; SBP, systolic blood pressure; LVEF, left ventricular ejection fraction; CK, creatine kinase; CK-MB, creatine kinase isoenzymes B; LDH, lactate dehydrogenase; HBDH, hydroxybutyrate dehydrogenase; HDL, high density lipoprotein; LDL, low density lipoprotein; VLDL, very low density lipoprotein; WBC, white blood cell; Hb, hemoglobin. CR time, the time from Symptom to CR (≥3 days and <3 days); admission time, the time from Symptom to admission (≥1 day and <1 day).
