# Supplementary material for: Factors related to cardiac rupture after acute myocardial infarction
Source: Front Cardiovasc Med. 2024 Oct 2;11:1401609. doi: 10.3389/fcvm.2024.1401609 (PMC11479954; doi:10.3389/fcvm.2024.1401609)
Supplement: Supplementary file 1 [file Datasheet1.zip › Supplementary Material/Table 7.docx]

Table 8. Odds ratios and 95% CIs for the association between different gender and CR risk.

| **Variables** | **Female** | | **Male** | |
| --- | --- | --- | --- | --- |
|  | **OR（95%CI）** | ***P* value** | **OR（95%CI）** | ***P* value** |
| Age | 1.096(1.002, 1.197) | 0.044* | 0.970(0.907, 1.038) | 0.379 |
| SBP | 0.936(0.899, 0.975) | 0.002* | 1.008(0.984, 1.033) | 0.505 |
| BMI | 0.996(0.804, 1.233) | 0.968 | 0.943(0.787, 1.129) | 0.522 |
| cTnI | 1.000(0.982, 1.018) | 0.974 | 1.051(1.023, 1.079) | <0.001** |
| LDH | 0.998(0.995, 1.000) | 0.101 | 0.999(0.997, 1.000) | 0.062 |
| Neut% | 1.139(1.029, 1.260) | 0.012* | 1.108(1.025, 1.199) | 0.010* |
| Albumin | 0.841(0.687, 1.030) | 0.094 | 0.880(0.743, 1.043) | 0.141 |
| Creatinine | 0.975(0.950, 1.001) | 0.060 | 1.007(0.996, 1.018) | 0.197 |
| Triglyceride | 0.508(0.119, 2.164) | 0.359 | 0.169(0.032, 0.896) | 0.037* |
| Bicarbonate | 0.775(0.612, 0.983) | 0.035* | 0.966(0.825, 1.132) | 0.672 |
| MI location (anterior MI) | 5.977 (1.025, 34.838) | 0.047* | 11.761(2.651, 52.177) | 0.001** |
| Killip class (>II) | 13.400(1.146, 156.652) | 0.039* | 11.901(2.273, 62.314) | 0.003* |

CR, cardiac rupture; MI, myocardial infarction; BMI, body mass index; SBP, systolic blood pressure; cTnI, cardiac troponin I; LDH, lactate dehydrogenase; Neut%, neutrophil percentage; OR, odds ratio; CI, confidence interval. **P* < 0.05; ***P* ≤ 0.001.
