# Supplementary material for: Factors related to cardiac rupture after acute myocardial infarction
Source: Front Cardiovasc Med. 2024 Oct 2;11:1401609. doi: 10.3389/fcvm.2024.1401609 (PMC11479954; doi:10.3389/fcvm.2024.1401609)
Supplement: Supplementary file 1 [file Datasheet1.zip › Supplementary Material/table 6.docx]

| Variable | Unadjusted OR（95%CI） | *P* value | Adjusted OR（95%CI） | *P* value |
| --- | --- | --- | --- | --- |
| Sex | 1.778 (0.947, 3.339) | 0.073 |  |  |
| Age | 1.057 (1.026, 1.090) | <0.001** |  |  |
| Hypertension | 1.217 (0.658, 2.250) | 0.531 | 1.076 (0.568, 2.038) | 0.822 |
| Cerebral infarction | 0.888 (0.367, 2.15) | 0.792 | 0.732 (0.294, 1.824) | 0.503 |
| Previous MI | 0.545 (0.156, 1.902) | 0.341 | 0.563 (0.157, 2.024) | 0.379 |
| BMI | 0.892 (0.811, 0.982) | 0.020* | 0.920 (0.834, 1.016) | 0.100 |
| SBP | 0.978 (0.964, 0.992) | 0.003* | 0.979 (0.965, 0.993) | 0.004* |
| DBP | 0.988 (0.969, 1.008) | 0.242 | 0.995(1.020, 1.086) | 0.660 |
| DM | 1.026 (0.507, 2.077) | 0.942 | 0.889 (0.423, 1.870) | 0.575 |
| LVEF | 0.943 (0.907, 0.981) | 0.004* | 0.953 (0.915, 0.994) | 0.023* |
| cTnI | 1.012 (1.002, 1.022) | 0.023* | 1.011 (1.001, 1.021) | 0.026* |
| MYO | 1.000 (1.000, 1.001) | 0.213 | 1.000 (1.000, 1.001) | 0.282 |
| CK | 1.000 (1.000, 1.000) | 0.182 | 1.000 (1.000, 1.000) | 0.437 |
| CK-MB | 1.000 (0.998, 1.002) | 0.749 | 1.000 (0.998, 1.002) | 0.873 |
| LDH | 1.001 (1.000, 1.001) | 0.093 | 1.001 (1.000, 1.001) | 0.071 |
| HBDH | 1.001 (1.000, 1.001) | 0.165 | 1.001 (1.000, 1.001) | 0.090 |
| Glucose | 1.085 (0.992, 1.187) | 0.074 | 1.074 (0.978, 1.180) | 0.137 |
| WBC | 1.277 (1.164, 1.402) | <0.001** | 1.296 (1.173, 1.433) | <0.001** |
| RBC | 0.747 (0.451, 1.237) | 0.257 | 1.089 (0.622, 1.907) | 0.766 |
| Hb | 0.992 (0.976, 1.008) | 0.325 | 1.004 (0.986, 1.023) | 0.659 |
| PLT | 1.002 (0.997, 1.006) | 0.458 | 1.002 (0.998, 1.006) | 0.315 |
| Neut% | 1.097 (1.052, 1.144) | <0.001** | 1.091 (1.046, 1.137) | <0.001** |
| TP | 0.956 (0.914, 1.019) | 0.200 | 0.981 (0.928, 1.038) | 0.511 |
| AIB | 0.874 (0.808, 0.946) | 0.001** | 0.904 (0.832, 0.981) | 0.016* |
| Cr | 1.007 (1.002, 1.012) | 0.009* | 1.007 (1.001, 1.012) | 0.016* |
| HDL | 1.225 (0.522, 2.872) | 0.641 | 1.111 (0.436, 2.830) | 0.826 |
| LDL | 1.058 (0.706, 1.585) | 0.785 | 1.177 (0.761, 1.820) | 0.464 |
| VLDL | 0.327 (0.115, 0.931) | 0.036* | 0.435(0.144, 1.313) | 0.140 |
| LP(a) | 1.001 (1.000, 1.002) | 0.248 | 1.001 (1.000, 1.002) | 0.153 |
| TG | 0.404 (0.213, 0.766) | 0.005* | 0.443 (0.219, 0.897) | 0.024* |
| TC | 0.913 (0.657, 1.269) | 0.588 | 0.996 (0.690, 1.436) | 0.981 |
| Bicarbonate | 0.910 (0.837, 0.990) | 0.028* | 0.931 (0.855, 1.014) | 0.100 |
| MI location (anterior MI) | 3.255 (1.692, 6.261) | <0.001** | 3.689 (1.850, 7.355) | <0.001** |
| Killip class≥ II | 6.977 (3.252, 14.968) | <0.001** | 5.946 (2.709, 13.051) | <0.001** |

Table 6. adjusted and Unadjusted univariate logistic regression analysis for the CR

(adjustment factors: age, sex). CR, cardiac rupture; Cerebral infarction, previous cerebral infarction; MI, myocardial infarction; BMI, body mass index; DBP, diastolic blood pressure; SBP, systolic blood pressure; DM, diabetes mellitus; LVEF, left ventricular ejection fraction; cTnI, cardiac troponin I; MYO, myoglobin; CK, creatine kinase; CK-MB, creatine kinase isoenzymes B; LDH, lactate dehydrogenase; HBDH, hydroxybutyrate dehydrogenase; WBC, white blood cell; RBC, red blood cell; Hb, Hemoglobin; PLT, Platelets; Neut%, neutrophil percentage; TP, total protein; ALB, albumin; Cr, creatinine; HDL, high density lipoprotein; LDL, Low Density Lipoprotein; VLDL, very low-density lipoprotein; LP(a), lipoprotein(a); TG, triglyceride; TC, total cholesterol. **P* < 0.05; ***P* **≤** 0.001.
