# Supplementary material for: Factors related to cardiac rupture after acute myocardial infarction
Source: Front Cardiovasc Med. 2024 Oct 2;11:1401609. doi: 10.3389/fcvm.2024.1401609 (PMC11479954; doi:10.3389/fcvm.2024.1401609)
Supplement: Supplementary file 1 [file Datasheet1.zip › Supplementary Material/table 8.docx]

Table 7. Univariate COX regression analysis for CR

| Variables | HR | 95% CI | *P* value |
| --- | --- | --- | --- |
| Age | 1.055 | 1.005-1.106 | 0.030* |
| Sex | 0.498 | 0.232-1.068 | 0.073 |
| BMI | 0.975 | 0.476-2.313 | 0.683 |
| Previous MI | 0.640 | 0.151-2.710 | 0.545 |
| Cerebral infarction | 2.767 | 1.097-6.984 | 0.031* |
| SBP | 0.992 | 0.978-1.006 | 0.250 |
| DBP | 0.981 | 0.961-1.001 | 0.067 |
| Hypertension | 1.054 | 0.501-2.216 | 0.891 |
| DM | 0.711 | 0.301-1.680 | 0.437 |
| cTnI | 0.999 | 0.989-1.009 | 0.853 |
| MYO | 1.000 | 0.998-1.001 | 0.486 |
| CK | 1.000 | 1.000-1.001 | 0.179 |
| CK-MB | 1.000 | 0.999-1.002 | 0.622 |
| LDH | 1.000 | 0.999-1.001 | 0.405 |
| HBDH | 1.001 | 1.000-1.001 | 0.210 |
| Glucose | 0.953 | 0.843-1.077 | 0.441 |
| WBC | 0.946 | 0.863-1.038 | 0.242 |
| Neut% | 0.974 | 0.928-1.023 | 0.296 |
| RBC | 1.225 | 0.700-2.144 | 0.478 |
| Hb | 1.005 | 0.988-1.023 | 0.543 |
| PLT | 1.001 | 0.997-1.005 | 0.620 |
| Cr | 0.992 | 0.982-1.002 | 0.133 |
| TP | 0.984 | 0.923-1.048 | 0.611 |
| Albumin | 0.966 | 0.897-1.041 | 0.364 |
| Lipoprotein(a) | 1.002 | 1.001-1.003 | 0.002* |
| LVEF | 1.003 | 0.953-1.054 | 0.922 |
| TG | 1.173 | 0.577-2.384 | 0.660 |
| TC | 0.994 | 0.665-1.485 | 0.975 |
| HDL | 0.718 | 0.205-2.513 | 0.605 |
| LDL | 0.987 | 0.612-1.593 | 0.959 |
| VLDL | 1.344 | 0.312-5.793 | 0.692 |
| Bicarbonate | 1.032 | 0.947-1.126 | 0.470 |
| Infarct location (anterior MI) | 1.049 | 0.476-2.313 | 0.905 |
| Primary PCI | 0.860 | 0.379-1.954 | 0.719 |
| Site of CR |  |  | 0.001** |
| VSR | 0.191 | 0.081-0.451 | <0.001** |
| PMR | 0.351 | 0.102-1.206 | 0.374 |
| CR time≥3d | 0.430 | 0.206-0.897 | 0.024* |
| Admission time <1d | 0.865 | 0.393-1.903 | 0.718 |
| Beta-blockers | 0.630 | 0.268-1.479 | 0.289 |
| ACEI | 0.918 | 0.433-1.949 | 0.825 |
| Heparins | 0.510 | 0.221-1.173 | 0.113 |

CR, cardiac rupture; BMI, body mass index; MI, myocardial infarction; Cerebral infarction, previous cerebral infarction; SBP, systolic blood pressure; DBP, diastolic blood pressure; DM, diabetes mellitus; cTnI, cardiac troponin I; MYO, myoglobin; CK, creatine kinase; CK-MB, creatine kinase isoenzymes B; LDH, lactate dehydrogenase; HBDH, hydroxybutyrate dehydrogenase; WBC, white blood cell; Neut%, neutrophil percentage; RBC, red blood cell; Hb, Hemoglobin; PLT, Platelets; Cr, creatinine; TP, total protein; LVEF, left ventricular ejection fraction; TG, triglyceride; TC, total cholesterol; HDL, high density lipoprotein; LDL, Low Density Lipoprotein; VLDL, very low-density lipoprotein; PCI, percutaneous coronary intervention; VSR, ventricular septal rupture; PWR, papillary muscle rupture; ACEI, Angiotensin-Converting Enzyme Inhibitors. CR time, the time from Symptom to CR; admission time, the time from Symptom to admission. **P* < 0.05; ***P* **≤** 0.001.
